# Supplementary material for: OPTO-BLUE: An Integrated Bidirectional Optogenetic Lentiviral Platform for Controlled Light-Induced Gene Expression
Source: Int J Mol Sci. 2023 May 31;24(11):9537. doi: 10.3390/ijms24119537 (PMC10253389; doi:10.3390/ijms24119537)
Supplement: Supplementary file 1 [file ijms-24-09537-s001.zip › ijms-2402390-supplementary.pdf]

Supplemental Information for

OPTO-BLUE: An integrated bidirectional optogenetic lentiviral platform for controlled light-induced gene expression

Duxan Arancibia [1], Iracy Pol [1], Martín Vargas-Fernández [1], Rafaella V. Zárate [1,2], Janetti R. Signorelli [1] and Pedro Zamorano [1,2,*]

This PDF file includes:

Supplemental figure S1

Title and legend for Supplemental figure S1

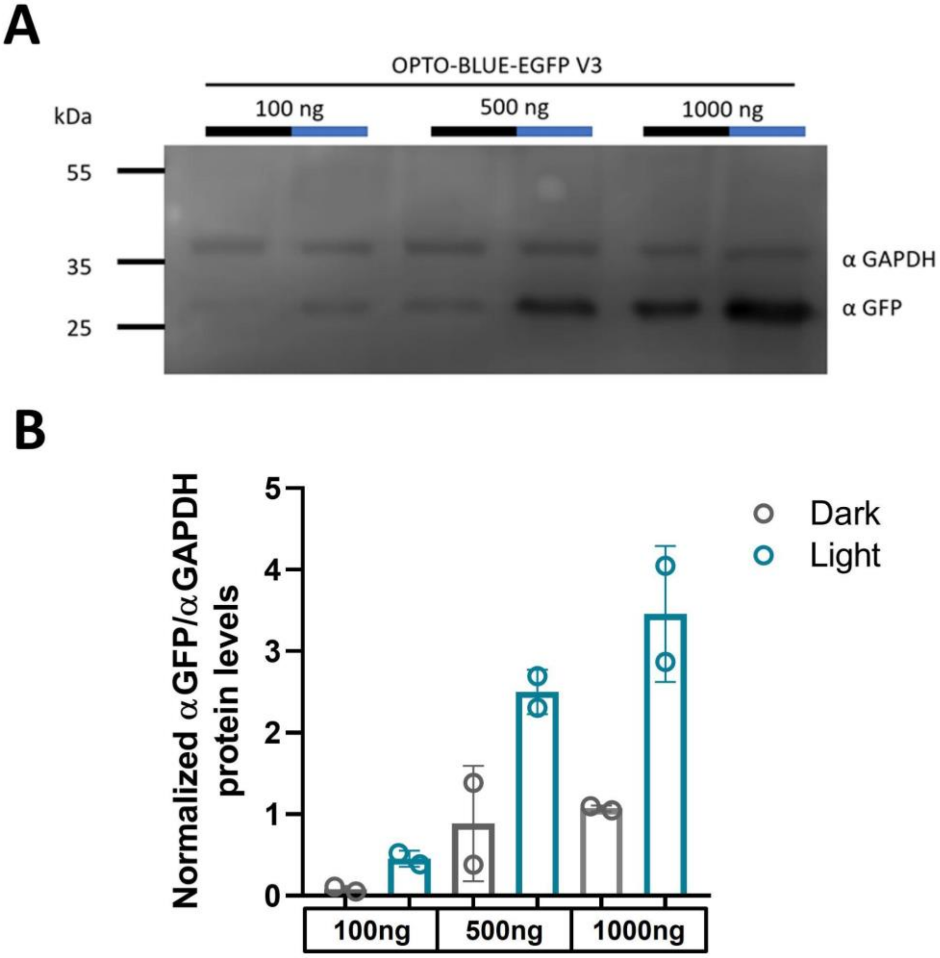

Supplementary Figure S1. Effect of the pOPTO-BLUE-EGFP-V3_500bp vector concentration (ng) on the blue light-mediated EGFP induction in HEK293-T cells. (A) HEK293-T cells were transfected with 100 ng, 500 ng, or 1000 ng of optogenetic lentiviral vector pOPTO-BLUE-EGFP-V3_500bp and subsequently, immunodetection of EGFP and GAPDH was performed. (B) Quantification of EGFP bands was performed by densitometry as indicated in the material and methods. The intensity of EGFP bands was normalized with the intensity of GAPDH bands (loading control).
